# Enhanced UVC Responsivity of Heteroepitaxial α-Ga_2_O_3_ Photodetector with Ultra-Thin HfO_2_ Interlayer

**DOI:** 10.3390/mi16070836

**Published:** 2025-07-21

**Authors:** SiSung Yoon, SeungYoon Oh, GyuHyung Lee, YongKi Kim, SunJae Kim, Ji-Hyeon Park, MyungHun Shin, Dae-Woo Jeon, GeonWook Yoo

**Affiliations:** 1 Department of Intelligent Semiconductor, Soongsil University, Seoul 06938, Republic of Korea; yss0615@gmail.com (S.Y.); osy1549@gmail.com (S.O.); lkh5057@naver.com (G.L.); 2 Department of Electronics and Information Engineering, Korea Aerospace University, Goyang 10540, Republic of Korea; ygk1740@gmail.com (Y.K.); mhshin@kau.ac.kr (M.S.); 3 Korea Institute of Ceramic Engineering & Technology, Jinju 52851, Republic of Korea; kau.sjkim@gmail.com (S.K.); jhp5511@kicet.re.kr (J.-H.P.); dwjeon@kicet.re.kr (D.-W.J.)

**Keywords:** α-Ga_2_O_3_, UVC photodetectors, hafnium oxide, metal–semiconductor–metal, responsivity

## Abstract

In this study, the influence of HfO_2_ interlayer thickness on the performance of heteroepitaxial α-Ga_2_O_3_ layer-based metal–insulator–semiconductor–insulator–metal (MISIM) ultraviolet photodetectors is examined. A thin HfO_2_ interlayer enhances the interface quality and reduces the density of interface traps, thereby improving the performance of UVC photodetectors. The fabricated device with a 1 nm HfO_2_ interlayer exhibited a significantly reduced dark current and higher photocurrent than a conventional metal–semiconductor–metal (MSM). Specifically, the 1 nm HfO_2_ MISIM device demonstrated a photocurrent of 2.3 μA and a dark current of 6.61 pA at 20 V, whereas the MSM device exhibited a photocurrent of 1.1 μA and a dark current of 73.3 pA. Furthermore, the photodetector performance was comprehensively evaluated in terms of responsivity, response speed, and high-temperature operation. These results suggest that the proposed ultra-thin HfO_2_ interlayer is an effective strategy for enhancing the performance of α-Ga_2_O_3_-based UVC photodetectors by simultaneously suppressing dark currents and increasing photocurrents and ultimately demonstrate its potential for stable operation under extreme environmental conditions.

## 1. Introduction

Ultraviolet (UV) detection is crucial for a wide range of applications, including fire detection, sterilization, environmental monitoring, aerospace, and defense systems. Within the UV spectrum, high-energy UVC radiation (wavelength < 280 nm) is particularly significant in extreme environments [1,2,3,4,5]. However, its carcinogenic nature and capacity to cause severe DNA damage pose significant risks to biological systems, including human skin and eyes [6,7]. Although the stratospheric ozone layer effectively absorbs most UVC radiation, the selective detection of weak UVC signals under visible or solar illumination remains a considerable technical challenge. Therefore, the development of highly sensitive and selective UVC detectors capable of operating in solar environments is essential for advancing these critical applications.

Conventional wide-bandgap semiconductors, such as aluminum gallium nitride (AlGaN) and diamond, have long been considered for UVC detection. Yet, AlGaN faces challenges in achieving high-quality thin films because of the complexities of its ternary alloy system, whereas diamond, despite its exceptional intrinsic properties, is hindered by its high fabrication cost and limited scalability for large-area integration [8,9,10,11,12].

These limitations have spurred interest in gallium oxide (Ga_2_O_3_), an ultra-wide-bandgap material (4.6–5.3 eV) emerging as a next-generation candidate for UVC photodetectors. Ga_2_O_3_ exists in five different crystal phases (α, β, γ, δ, and ε), among which the α-phase exhibits the widest bandgap (~5.2 eV) and a nearly direct band-to-band transition. In addition, α-Ga_2_O_3_ can be grown at relatively low temperatures (~500 °C), which is advantageous for industrial-scale manufacturing. Combined with its visible-blind nature that effectively suppresses noise from visible and near-UV light, α-Ga_2_O_3_ also offers low-noise and high-sensitivity performance, making it a highly promising material for reliable and highly selective UVC photodetectors, particularly in extreme environments, such as flame detection, where precision and stability are essential [13,14,15,16,17]. Furthermore, α-Ga_2_O_3_ offers excellent compatibility with large-area sapphire substrates and can be grown using various techniques, including hydride vapor phase epitaxy (HVPE), mist chemical vapor deposition (mist-CVD), and molecular beam epitaxy (MBE). Among these, HVPE is particularly favorable for scalable production due to its extremely high growth rate, while MBE, though capable of producing highly crystalline films, is constrained by a slow deposition rate that limits its suitability for mass production [18,19,20,21]. These attributes make α-Ga_2_O_3_ a promising material for high-reliability, selective UVC photodetectors, particularly in demanding applications, such as flame detection, where precision and stability are paramount [22,23,24].

In this study, α-Ga_2_O_3_-based metal–insulator–semiconductor–insulator–metal (MISIM) UVC photodetectors were fabricated and systematically characterized. We fabricated devices with different HfO_2_ thicknesses to explore their impact, systematically evaluating performance through current–voltage (*I–V*) measurements under dark and UVC-illuminated conditions. These tests revealed significant dark current suppression and photocurrent enhancement. A spectral responsivity analysis at 235 nm confirmed the high wavelength selectivity and rejection ratio of the fabricated device. In addition, time-dependent photocurrent measurements were used to assess the response speed, and high-temperature measurements up to 300 °C were conducted to evaluate thermal stability. These results demonstrate that the incorporation of a thin HfO_2_ interlayer effectively enhances the photoresponse and reliability of α-Ga_2_O_3_-based UVC photodetectors, thereby supporting their practical applicability in extreme and high-temperature environments.

## 2. Experimental Section

Heteroepitaxial α-Ga_2_O_3_ layers were grown on a 2-inch (0001) sapphire substrate using hydride vapor phase epitaxy (HVPE) at 470 °C, with GaCl and O_2_ as precursors. The undoped α-Ga_2_O_3_ films were grown to a thickness of 700 nm for 6 min. Following surface cleaning with acetone and isopropyl alcohol (IPA), HfO_2_ layers were deposited via atomic layer deposition (ALD) at 360 °C, using tetrakis (ethylmethylamino) hafnium (TEMAhf) as the precursor and ozone as the reactant. HfO_2_ thicknesses of 1 nm and 2 nm were achieved, with a growth rate of approximately 0.74 Å/cycle. Interdigitated Ti/Au (20 nm/80 nm) electrodes were then fabricated using electron-beam evaporation and a lift-off process, covering an active area of 0.5 mm^2^, with electrode widths of 5 μm and an interelectrode spacing of 12.5 μm. Two device configurations were prepared: a metal–insulator–semiconductor–insulator–metal (MISIM) structure with the HfO_2_ insulating layer and a metal–semiconductor–metal (MSM) structure without it.

For UVC illumination, a monochromator (CS 130B-1-MC, Newport Corporation, Irvine, CA, USA) paired with a xenon arc lamp (6269, Newport Corporation, USA) was used, and the wavelength-specific intensity was measured with an optical power and energy meter (2938-R, Newport Corporation, USA). Current–voltage (*I–V*) characteristics were assessed using a precision source/measurement unit (B2912B, Keysight, Santa Rosa, CA, USA). The crystal quality was analyzed by high-resolution X-ray diffraction (HR-XRD) on a SmartLAB system (Rigaku), and the surface chemistry was characterized via X-ray photoelectron spectroscopy (XPS) using a VersaProbe III system (ULVAC-PHI). A transmission electron microscope (TEM) was used for the crystal structure (EM-ARM200F, JEOL, Ltd., Tokyo, Japan) at 200 keV. The selected area electron diffraction (SAED) pattern was analyzed using the Gatan Digital Micrograph^®^ software (version 3.6.0, AMETEK, Inc., Berwyn, PA, USA).

## 3. Results

**Figure 1:**
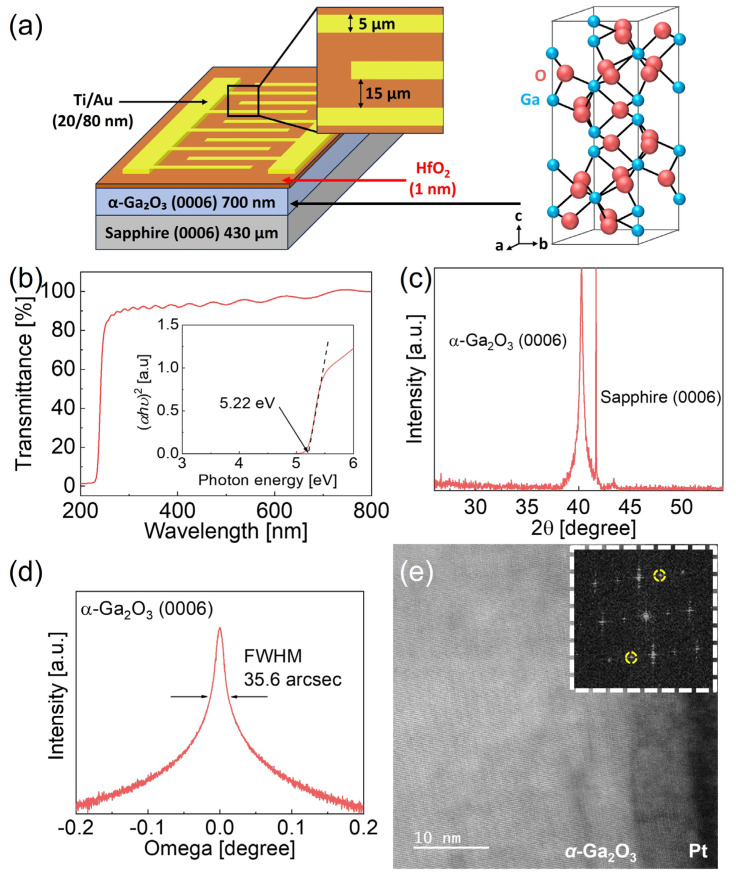
(**a**) Schematic of the MISIM photodetector, featuring an HfO_2_ insulating layer on α-Ga_2_O_3_ and a 3D schematic diagram of the α-Ga_2_O_3_ lattice structure. (**b**) Optical transmittance spectrum with an inset showing the optical bandgap of the heteroepitaxial α-Ga_2_O_3_ layer, estimated using a Tauc plot. (**c**) High-resolution X-ray diffraction (HR-XRD) 2θ scan of the heteroepitaxial α-Ga_2_O_3_ layer. (**d**) HR-XRD rocking curve for the (0006) diffraction peak. (**e**) Cross-view transmission electron microscope (TEM) image of α-Ga_2_O_3_ thin film with an inset selected area electron diffraction pattern.

Figure 1a illustrates a schematic of the fabricated device and a 3D schematic diagram of the α-Ga_2_O_3_ lattice structure. Figure 1b presents the optical transmittance spectrum and Tauc plot of the heteroepitaxial α-Ga_2_O_3_ layer, used to determine its optical bandgap. The results show that the α-Ga_2_O_3_ film fully absorbs light with wavelengths below 250 nm, and its optical bandgap was calculated to be approximately 5.22 eV, derived from the Tauc plot using Equation (1).

(*αhν*)^2^ = *A*(*hν* − *Eg*) (1)

This value is consistent with the previously reported bandgaps of α-Ga_2_O_3_ [18,20,22]. In addition, Figure 1c presents the XRD measurements, revealing two distinct diffraction peaks corresponding to the Al_2_O_3_ (0006) and α-Ga_2_O_3_ (0006) planes. These findings confirm that the α-Ga_2_O_3_ film grew epitaxially along the (0006) direction on the sapphire (Al_2_O_3_) substrate. In Figure 1d, the rocking curve of α-Ga_2_O_3_ exhibits a full width at half maximum (FWHM) of 35.6 arcseconds, which is consistent with previously reported values for α-Ga_2_O_3_ [22,25].

The cross-view TEM image in Figure 1e shows that α-Ga_2_O_3_ was preferentially grown in the (0006) direction along the c-axis of the sapphire substrate, consistent with the XRD results. The ideal d-spacing of α-Ga_2_O_3_ (0006) is 2.238 Å, whereas the measured d-spacing from the SAED pattern is 2.215 Å, showing evidence of compressive strain. This has been attributed to the residual stresses caused by the lattice mismatch between the sapphire substrate and heteroepitaxial α-Ga_2_O_3_ layer, which is generally observed in a heteroepitaxial growth of α-Ga_2_O_3_ on a sapphire substrate [26].

**Figure 2:**
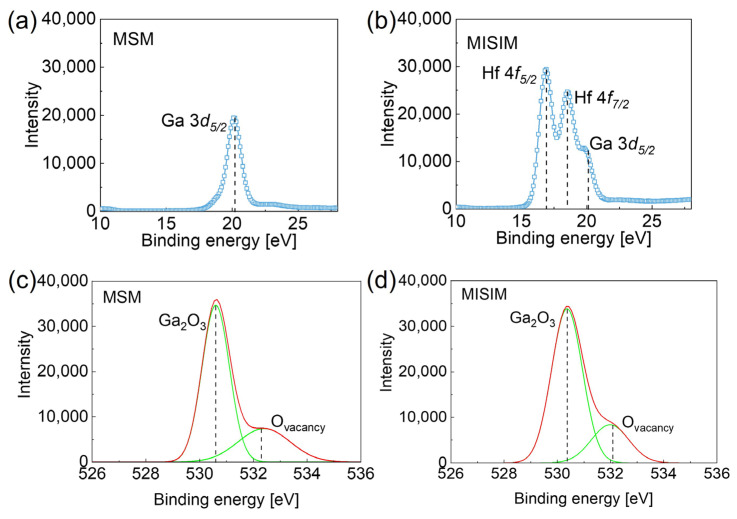
(**a**) XPS spectrum of Ga 3*d*_5*/*2_ for the MSM structure; (**b**) XPS spectra of Hf 4*f*_5*/*2_, Hf 4*f*_7*/*2_, and Ga 3*d*_5*/*2_ for the MISIM structure. The O 1s XPS spectrum (**c**) for the MSM structure and (**d**) for the MISIM structure, showing Ga_2_O_3_ and oxygen vacancies.

Figure 2 investigates the deposition of HfO_2_ via X-ray photoelectron spectroscopy (XPS) and assesses the impact of ozone supplied during atomic layer deposition (ALD) on oxygen vacancies. Figure 2a presents the Ga 3*d* spectrum for the MSM structure, showing a peak at a binding energy of 20.1 eV. In contrast, Figure 2b reveals additional Hf 4*f* peaks at 16.9 eV and 18.5 eV in the MISIM structure, confirming successful HfO_2_ deposition [27,28]. Figure 2c,d display the O 1s spectra, deconvoluted into two peaks at 530.6 eV (Ga-O bonds) and 532.1 eV (oxygen vacancies). The oxygen vacancy ratio, calculated from the peak areas, was 28.8% for the MSM structure and 23.1% for the MISIM structure, indicating a 5.7% reduction attributed to the oxygen vacancy treatment effect from the ozone during ALD. Oxygen vacancies are known to impair photodetector performance by increasing the dark current [29]. While oxygen annealing typically requires temperatures above 600 °C for a significant effect, the ALD process at 360 °C yielded only modest vacancy reduction [30,31].

**Figure 3:**
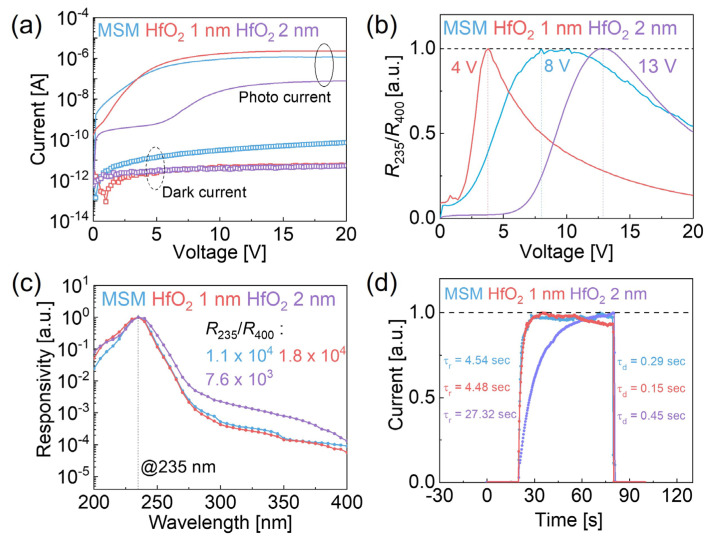
(**a**) Dark current and photocurrent under 235 nm UVC illumination for MSM and MISIM devices with 1 nm and 2 nm HfO_2_ layers. (**b**) Responsivity rejection ratio (R_235_/R_400_) as a function of voltage. (**c**) Responsivity as a function of wavelength at the peak voltage. (**d**) Response speed during UVC on/off switching at 235 nm for MSM, 1 nm HfO_2_ MISIM, and 2 nm HfO_2_ MISIM devices.

Figure 3a presents the *I–V* characteristics of the α-Ga_2_O_3_ MSM and MISIM devices under dark and UVC illumination (235 nm) conditions. At 20 V, the dark currents were 73.3 pA for the MSM device, 6.61 pA for the MISIM device with 1 nm HfO_2_, and 5.27 pA for the 2 nm HfO_2_ device, indicating that HfO_2_ layers effectively reduce the dark current. The decrease in the dark current is attributed to suppressed electron injections due to reduced tunneling through the additional insulating HfO_2_ layer. Under UVC illumination at 20 V, the photocurrents were 1.14 μA (MSM), 2.3 μA (1 nm HfO_2_ MISIM), and 79.4 nA (2 nm HfO_2_ MISIM), with the 1 nm HfO_2_ MISIM device surpassing the MSM device. The observed enhanced photocurrent is presumably attributed to reduced trap states at the HfO_2_/α–Ga_2_O_3_ interface, not due to a change in Schottky barrier height. The 2 nm HfO_2_ MISIM device showed an unstable photocurrent below 8 V, attributed to trap states at the HfO_2_/Ga_2_O_3_ interface, which immobilize electrons in the Ga_2_O_3_ layer at low voltages. However, at voltages above 8 V, the traps become sufficiently filled, allowing electrons to escape under UVC illumination, resulting in an increased current [32,33]. Figure 3b compares the responsivity rejection ratio as a function of the applied voltage at wavelengths of 235 and 400 nm, highlighting the peak voltages. Figure 3c compares these wavelengths under the peak voltage, calculated using Equation (2):

(2) $$R=(I_{p}-I_{d})/\;P\times S_{\mathrm{device}}$$

where *I_p_* is the channel current under illumination, *I_d_* is the dark current, *P* is the incident light intensity, and *S_device_* is the effective irradiated area. The rejection ratios (R_235_/R_400_) were 1.1 × 10^4^ (MSM), 1.8 × 10^4^ (1 nm HfO_2_ MISIM), and 7.6 × 10^3^ (2 nm HfO_2_ MISIM), with the 1 nm HfO_2_ MISIM device showing high responsivity at 235 nm and low responsivity at 400 nm, confirming enhanced UVC selectivity. Figure 3d shows the response current during UVC switching on/off at 235 nm under a bias of 20 V. The rise time (*τ_r_*) and decay time (*τ_d_*), defined as the time required for the current to change from 10% to 90% and from 90% to 10% of its final value, respectively, were measured for the MSM, as well as the 1 and 2 nm HfO_2_ MISIM devices as 4.51 s, 4.48 s, and 27.32 s for *τ_r_* and 0.29 s, 0.15 s, and 0.45 s for *τ_d_*, respectively. The 1 nm HfO_2_ MISIM device’s faster *τ_r_* and *τ_d_* resulted from the reduced trapping of photogenerated holes at the surface states. Conversely, the 2 nm HfO_2_ MISIM device’s thicker layer lowered the tunneling probability, slowing carrier transport and extending response times [34].

**Figure 4:**
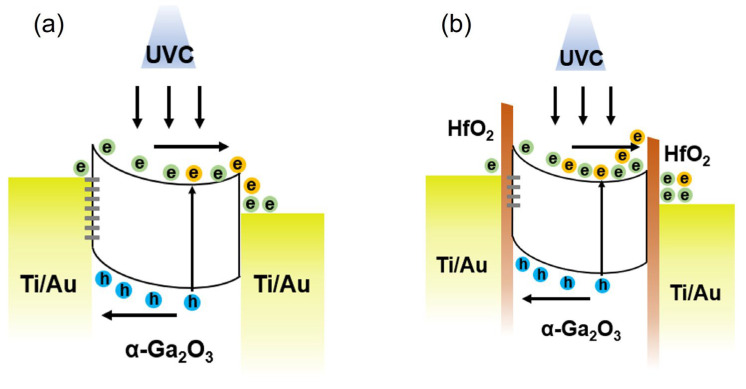
Energy band diagrams of heteroepitaxial α-Ga_2_O_3_ layer-based UVC photodetectors: (**a**) MSM structure and (**b**) MISIM structure.

Figure 4a,b show the band diagrams of the MSM and MISIM structures, respectively. Typically, an insulating HfO_2_ layer suppresses tunneling, reducing the current, as evidenced by the lower dark currents in the MISIM devices shown in Figure 3a. However, when a 1 nm HfO_2_ layer is used under UVC illumination (energy exceeding the α-Ga_2_O_3_ bandgap), the MISIM device exhibits an increased photocurrent. As shown in Figure 4b, the HfO_2_/Ga_2_O_3_ interface in the MISIM structure has fewer traps than the Ga_2_O_3_/Ti/Au interface in the MSM structure, enhancing the photocurrent by mitigating trap-related losses. This improvement is expected to outweigh the current reduction owing to tunneling suppression [35,36]. However, increasing the HfO_2_ thickness to 2 nm reduces the photocurrent, as the lower tunneling probability dominates over the benefits of reduced traps. For a dark current, where no UVC energy is present, even a 1 nm HfO_2_ layer effectively suppresses tunneling, resulting in a significantly lower dark current in the MISIM structure compared to the MSM structure.

**Figure 5:**
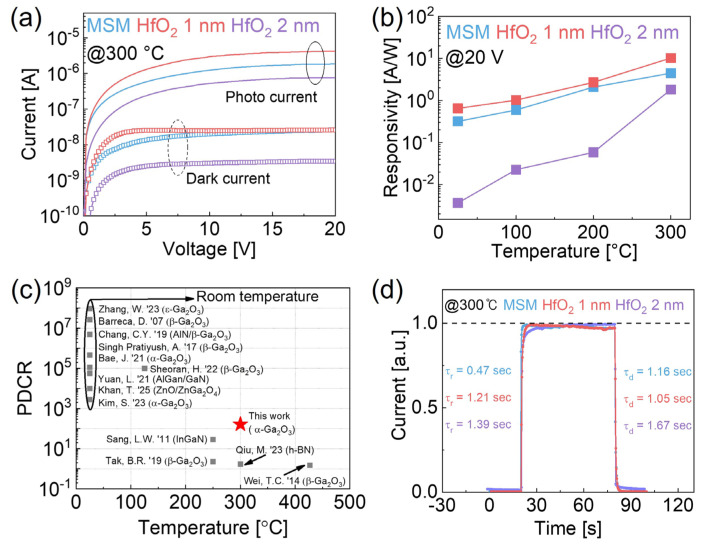
(**a**) Photocurrent and dark current at 300 °C for MSM and MISIM devices with 1 nm and 2 nm HfO_2_ layers. (**b**) Responsivity at 20 V as a function of temperature; (**c**) benchmark plot of PDCR for variable high temperatures [3,4,10,15,19,22,28,31,33,38,39,40]; (**d**) response speed during UVC on/off switching at 235 nm and 300 °C for the MSM, 1 nm HfO_2_, and 2 nm HfO_2_ devices.

Figure 5a presents the *I–V* measurements at 300 °C. Below 8 V, the 1 nm HfO_2_ MISIM device exhibited a higher current than the MSM device, which is attributed to reduced interface traps and moderated tunneling suppression. Figure 5b compares the responsivity at 20 V as a function of temperature, showing an increase with temperature. This behavior can be attributed to thermal excitation, which affects both *I_p_* and *I_d_*. However, while the increased *I_d_* is primarily attributed to thermally generated carriers, the increased *I_p_* is ascribed not only to the thermally generated carriers but also to enhanced photoexcitation at elevated temperatures, which further facilitates the de-trapping of carriers from trap states. Consequently, more carriers become available for conduction under illumination, leading to an increased photocurrent and enhanced responsivity at an elevated temperature [37]. Figure 5c illustrates PDCR with variable temperatures for UV photodetectors based on Ga_2_O_3_ and various other materials [3,4,10,15,19,22,28,31,33,38,39,40]. Most previously reported works have included characterizations at room temperature, and characterizations at elevated temperatures are not investigated enough. Our work demonstrates insertion of the ultra-thin HfO_2_ interlayer-enabled high PDCR even under high-temperature conditions. Figure 5d shows the transient response at 300 °C. The *τ_r_* and *τ_d_* for the MSM and 1 and 2 nm MISIM devices were 0.47 s, 1.21 s, and 1.39 s and 1.16 s, 1.05 s, and 1.67 s, respectively. At 300 °C, the increased thermal energy enabled electrons in the MSM device to escape interface traps and enter the conduction band more readily, resulting in a shorter *τ_r_* compared with the 1 nm HfO_2_ MISIM device. However, the decay times were longer than those at room temperature because of the increased persistent photoconductivity (PPC) effect. PPC refers to the phenomenon in which photoconductivity persists even after the light source has been removed. This effect is commonly observed in oxide semiconductors with oxygen vacancies and a high density of defects [3,37,41].

Table 1 summarizes the key performance metrics of Ga_2_O_3_ thin-film-based photodetectors, showing that the performance varies significantly depending on the Ga_2_O_3_ crystal phase and growth method [2,3,10,11,42,43,44]. The MISIM α-Ga_2_O_3_ device developed in this study demonstrated an enhanced photocurrent due to the reduced trap states at the HfO_2_/α-Ga_2_O_3_ interface and a suppressed dark current by the insulating HfO_2_ layer. These attributes resulted in both increased PDCR and rejection ratios that confirmed enhanced UVC selectivity. Furthermore, the use of the HVPE growth process presents strong potential for scalable production. Although further research is needed to improve film quality and device performance, this study offers a promising approach for the application of a heteroepitaxial α-Ga_2_O_3_ layer in UVC photodetectors.

## 4. Conclusions

In conclusion, this study investigated the impact of HfO_2_-layer presence and thickness on the optical and electrical performance of heteroepitaxial α-Ga_2_O_3_ layer-based UVC photodetectors. The structural and compositional characterizations of α-Ga_2_O_3_ were thoroughly analyzed using HR-XRD, XPS, TEM, and SAED. *I–V* measurements revealed that MISIM devices with 1 nm and 2 nm HfO_2_ layers achieved significantly lower dark currents than the MSM device, owing to the tunneling suppression induced by the HfO_2_ layer. Under 235 nm UVC illumination, the 1 nm HfO_2_ MISIM device delivered the highest photocurrent (2.3 μA at 20 V), driven by reduced surface traps, while the 2 nm HfO_2_ MISIM device showed a lower photocurrent due to increased tunneling resistance. In addition, the 1 nm HfO_2_ MISIM device exhibited the highest responsivity (1.8 × 10^4^ A/W), outperforming the MSM (1.1 × 10^4^ A/W) and 2 nm HfO_2_ MISIM (7.6 × 10^3^ A/W) devices, indicating enhanced UVC selectivity and a reduced UVC on/off response time. At 300 °C, the 1 nm HfO_2_ device exhibited a slight dark current increase from thermal-assisted tunneling but maintained superior responsivity compared to the MSM device. Overall, the 1 nm HfO_2_ layer optimized the photoresponse by minimizing traps and ensured robust performance at high temperatures, establishing α-Ga_2_O_3_-based MISIM photodetectors as highly effective for UVC detection in demanding environments.

## Figures and Tables

**Table 1:** Performance comparison of photodetectors fabricated on Ga_2_O_3_ thin films.

Phase	Grown	Detection	PDCR	Responsivity	Dark Current	Ref
Alpha	HVPE	235 nm	8.7 × 10^3^ (@20 V)	1.38 A/W (@8 V)	73 pA (@20 V)	This work (w/o)
Alpha	HVPE	235 nm	7.3 × 10^4^ (@20 V)	0.68 A/W (@4 V)	5.5 pA (@20 V)	This work (1 nm)
Alpha	HVPE	235 nm	5.2 × 10^2^ (@20 V)	0.17 A/W (@13 V)	5.2 pA (@20 V)	This work (2 nm)
Beta	PLD	254 nm	14 (@10 V)	30.45 A/W (@10 V)	4.2 nA	[2]
Beta	PLD	255 nm	7.1 × 10^3^ (@10 V)	0.74 A/W (@10 V)	0.32 nA	[3]
Beta	MBE	236 nm	10^3^ (@20 V)	1.5 A/W (@4 V)	4 nA (@20 V)	[10]
Beta	RF sputtering	254 nm	10^5^	303 A/W	10 pA (@20 V)	[11]
Amorphous	Sputtering	254 nm	2.6 × 10^7^ (@5 V)	3.2 × 10^4^ A/W (@ 5V)	51 pA (@5 V)	[42]
Epsilon	MOCVD	254 nm	2.4 × 10^7^	639 A/W (@10 V)	4.6 pA	[43]
Epsilon	MOCVD	252 nm	1.0 × 10^3^ (@15 V)	0.38 A/W (@15 V)	20 nA (@15 V)	[44]

## Data Availability

The data presented in this study are available on request from the corresponding author.
